# Calcium Ions Promote Formation of Amyloid β-Peptide (1–40) Oligomers Causally Implicated in Neuronal Toxicity of Alzheimer's Disease

**DOI:** 10.1371/journal.pone.0018250

**Published:** 2011-03-28

**Authors:** Anna Itkin, Vincent Dupres, Yves F. Dufrêne, Burkhard Bechinger, Jean-Marie Ruysschaert, Vincent Raussens

**Affiliations:** 1 Laboratory of Structure and Function of Biological Membranes, Center for Structural Biology and Bioinformatics, Université Libre de Bruxelles, Brussels, Belgium; 2 International Center for Frontier Research in Chemistry, Chemistry Institute, Membrane Biophysics and NMR, Université de Strasbourg, Strasbourg, France; 3 Institute of Condensed Matter and Nanosciences, Université Catholique de Louvain, Louvain-la-Neuve, Belgium; Federal University of Rio de Janeiro, Brazil

## Abstract

Amyloid β-peptide (Aβ) is directly linked to Alzheimer's disease (AD). In its monomeric form, Aβ aggregates to produce fibrils and a range of oligomers, the latter being the most neurotoxic. Dysregulation of Ca^2+^ homeostasis in aging brains and in neurodegenerative disorders plays a crucial role in numerous processes and contributes to cell dysfunction and death. Here we postulated that calcium may enable or accelerate the aggregation of Aβ. We compared the aggregation pattern of Aβ(1–40) and that of Aβ(1–40)E22G, an amyloid peptide carrying the Arctic mutation that causes early onset of the disease. We found that in the presence of Ca^2+^, Aβ(1–40) preferentially formed oligomers similar to those formed by Aβ(1–40)E22G with or without added Ca^2+^, whereas in the absence of added Ca^2+^ the Aβ(1–40) aggregated to form fibrils. Morphological similarities of the oligomers were confirmed by contact mode atomic force microscopy imaging. The distribution of oligomeric and fibrillar species in different samples was detected by gel electrophoresis and Western blot analysis, the results of which were further supported by thioflavin T fluorescence experiments. In the samples without Ca^2+^, Fourier transform infrared spectroscopy revealed conversion of oligomers from an anti-parallel β-sheet to the parallel β-sheet conformation characteristic of fibrils. Overall, these results led us to conclude that calcium ions stimulate the formation of oligomers of Aβ(1–40), that have been implicated in the pathogenesis of AD.

## Introduction

Alzheimer's disease (AD) is a progressive neurodegenerative disorder that affects nearly 2% of the population in industrialized countries. AD is the most common form of dementia and is characterized by brain cell destruction, memory loss, and deterioration of cognitive and behavioral processes severe enough to affect work, lifelong hobbies, and social life. Symptoms worsen over time and the disease is fatal.

For many years, the pathologic hallmark of AD was attributed to the continuous accumulation of amyloid β-peptide (Aβ) fibrils into plaques. Their toxic effects on synaptic connections and neurons were explained by the amyloid cascade hypothesis [1]. However, experiments aimed at establishing a direct causal relationship between Aβ deposition and the neurodegeneration that underlies AD dementia failed [2], [3]. This apparent discrepancy between plaque burden and neuronal dysfunction has been described in transgenic mouse models of AD [4], [5]. Recent theories that apparently resolve this inconsistency refer to small soluble oligomeric or protofibrillar assemblies of Aβ [6], [7], shown to be toxic to neurons and their vital interconnections [8]–[10]. Results of studies that focused on the electrophysiological impact of Aβ oligomers suggested that the underlying memory loss is caused by rapid inhibition of long-term potentiation (LTP)—a classical model for synaptic plasticity and memory mechanisms [11]—by oligomers [12]–[14], which might explain, at least in part, the mild cognitive impairment observed in the early stages of AD [9].

Aβ peptide is a product of the proteolytic cleavage of the amyloid precursor protein (APP). Although Aβ peptides may vary in length from 38 to 43 amino acids, the two main alloforms in the brain are Aβ(1–40) and Aβ(1–42). Both peptides have been found in amyloid plaques [15]–[17] and shown to form oligomers and protofibrils [18]. Post-mortem analysis in human subjects disclosed that Aβ(1–40) rather than Aβ(1–42), whether in soluble or in insoluble form, discriminated more readily between AD patients and high pathology controls [8]. In addition to sporadic cases of AD, several familial Alzheimer's disease (FAD) mutations have been discovered and studied over the years. Most of these mutations cause an increase in Aβ by interfering with APP processing. A new mutation within the APP sequence Aβ(E22G), found to cause AD in Swedish families, was reported in 2001 by Nilsberth et al., who named it the Arctic mutation. Those authors observed that carriers of this mutation showed decreased amounts of Aβ(1–42) and Aβ(1–40) in the plasma, and demonstrated that Aβ(1–40)E22G forms protofibrils much faster and more abundantly than the wild-type Aβ, whereas the rate of fibrillization remained the same [19]. Later studies suggested that the clinical and pathological features of patients with the Arctic mutation are attributable to increased generation of Aβ intermediates formed early in fibrillogenesis, as well as their greater stability [20]. Moreover, Aβ(1–40)E22G was shown to rapidly induce neurotoxicity, and that this correlated with the formation of small pre-fibrillar assemblies, including protofibrils [21]. Clinical and pathological features of FAD are indistinguishable from those of sporadic cases, but disease onset occurs at a much younger age in patients with the Arctic mutation [19], [22].

Research has so far failed to establish any unique primary mechanism underlying the Aβ aggregation followed by neuronal degeneration and death in patients with AD. Rather, it seems likely that numerous processes participate both in Aβ aggregation and in the ultimate development of the disease. One of the many hypotheses put forward to account for the etiology of AD argues that a central role in AD pathology is played by dysregulation of calcium homeostasis [23]–[25]. The idea that altered calcium homeostasis might serve as a trigger in the development of AD was first formulated in 1982 and later revised by Khachaturian [26].

The principal risk factor for AD is advanced age. In sporadic cases, the first manifestations of the disease symptoms occur towards the seventh decade of life. Neuronal activation is usually associated with an increase in intracellular Ca^2+^ concentration ([Ca^2+^]_i_), while the source of the Ca^2+^ ions can be either extracellular or intracellular. Age-related alterations in Ca^2+^-specific regulatory systems in neurons include increased amounts of intracellular Ca^2+^, enhanced Ca^2+^ influx through voltage-dependent Ca^2+^ channels, impaired mitochondrial ability to buffer or cycle Ca^2+^ [27], and perturbed Ca^2+^ regulation in ryanodine (Ry)-sensitive and Ins(1,4,5)P_3_-sensitive Ca^2+^ stores [28].

Numerous studies have implicated Ca^2+^ dysfunction in AD, demonstrating the bidirectional relationship between Ca^2+^ signaling and the amyloidogenic pathway [29], [30]. On the one hand, certain alterations in Ca^2+^ signaling are common to both sporadic and familial cases of AD [31], [32]. Direct measurements of [Ca^2+^]_i_ show that cells exposed to Aβ exhibit disruption in calcium homeostasis [33], [34], which may in turn cause a variety of secondary effects such as activation of cellular enzymes, induction of apoptosis, and cytoskeletal modifications [35], [36]. Aβ can reportedly trigger Ca^2+^ release from endoplasmic reticulum (ER) stores via interaction with IP_3_ and Ry receptors [37], [38], as well as an increase in calcium influx via the NMDA receptors [39], [40]. Formation of cation-selective channels by Aβ in bilayer membranes and in living cells [41], [42] further enhances the ability of this peptide to alter cytosolic Ca^2+^ homeostasis. On the other hand, changes in the amounts and dynamics of Ca^2+^ alter the metabolism and production of Aβ [30]. Influx of Ca^2+^ through calcium channels of the plasma membrane or through calcium release from ER stores increases Aβ generation [43]. An increase in cytosolic Ca^2+^ concentration, moreover, was shown to induce transient phosphorylation of APP and tau, leading to an increased production of intracellular Aβ [44].

In this study we used gel electrophoresis and Western blot analysis, thioflavine T (ThT) fluorescence, Fourier transform infrared spectroscopy (FTIR), and atomic force microscopy imaging (AFM) to compare the aggregation of Aβ(1–40) and Aβ(1–40)E22G, in order to study the structural and morphological similarities of the species—oligomeric or fibrillar—formed by both peptides in the presence and in the absence of added Ca^2+^. We postulated that when calcium dysregulation takes place under conditions of normal aging, it may facilitate the formation of pathogenic Aβ oligomers, which in turn may intensify the Ca^2+^ dyshomeostasis. The oligomeric species may then be held accountable for mediating the neuronal injury and LTP inhibition characteristic of AD in elderly individuals, similar to the situation in FAD patients carrying the Arctic mutation. Detailed knowledge of the action of Ca^2+^ upstream of Aβ is a prerequisite for complete understanding of the molecular mechanism(s) responsible for the age-related risks of Alzheimer's disease. Because Aβ plays a fundamental part in the development and devastating progression of AD, we believe that understanding of the formation and properties of its toxic forms will provide a key to the comprehension of the disease mechanism, thereby enabling us to develop novel preventive and therapeutic approaches.

## Methods

### Chemicals

All chemicals were purchased from Sigma-Aldrich or Bio-Rad, unless stated otherwise.

### Peptide preparation

The amyloid β-peptides Aβ(1–40) and Aβ(1–40)E22G were purchased from American Peptide Company in the form of lyophilized powder. Prior to use, 1 mg aliquots were dissolved in double-distilled water, sonicated in a water bath for 1 min, and then held in ice for 1 min. This cycle was repeated five times. The peptide solution was then divided into 50-μg aliquots and dried under vacuum in a ThermoSavant SpeedVac (UVS400A Universal Vacuum System). Aβ films were stored at −20°C until use.

### Sample preparation

The 50- µg aliquots of lyophilized Aβ peptide were hydrated at room temperature in either 50 mM phosphate buffer pH 7.4 and 100 mM NaCl (“–Ca^2+^ condition”) or 75 mM MOPS pH 7.4 and 2 mM CaCl_2_ (“+Ca^2+^ condition”). The “−Ca^2+^ condition” refers to the condition where no calcium was added to the buffer. However, contaminating calcium concentration was 21±1.3 µM, as was determined with inductively coupled plasma optical emission spectroscopy (ICP-OES). The final concentration of the peptide for all samples was 100 µM unless otherwise stated. Samples were sonicated for 2 min in a water bath and incubated at 37°C without agitation.

### Thioflavin T (ThT) fluorescence measurements

The thioflavin T (Sigma-Aldrich) fluorescence assay [45] was used to follow the aggregation profile of Aβ peptides for 96 h in an LS55 fluorimeter (Perkin Elmer Instruments). Aliquots of 10 µl (∼4 µg) of the incubated peptide solution were added to 1 ml of 50 mM glycine-NaOH buffer pH 8.5 and 5 µM ThT. The sample was maintained at 25°C in a circulating water bath. Excitation and emission wavelengths were 450 nm and 482 nm, respectively. The emission spectra were collected for 500 s. The intensity of each spectrum was then averaged over approximately 400 s, following subtraction of the averaged (100 s) background fluorescence.

### Western blot analysis

Peptide samples were diluted in a PAGE sample buffer and separated on a 12% bis-Tris gel at 4°C for 2 h at 100 V. There was no SDS in the acrylamide gel, but the sample buffer contained 4% SDS. Unboiled samples were loaded on the gel. The separated bands of protein were transferred onto a nitrocellulose membrane, which was then blocked for 1 h in 5% nonfat dry milk in Tris-buffered saline (TBS)/Tween-20 buffer. The membrane was incubated with a mixture of two mouse monoclonal Aβ antibodies, 6E10 (1∶3000) and 4G8 (1∶2000) (Sigma-Aldrich). Detection was carried out by enhanced chemiluminescence using horseradish peroxidase-conjugated goat anti-mouse antibody (1∶2000) (Boehringer Mannheim). Images were recorded and analyzed using the ImageQuant 400 gel imager and ImageQuant TL software (GE Healthcare).

### Fourier transform infrared (FTIR) spectroscopy

Infrared spectra were recorded on an Equinox 55 infrared spectrophotometer (Bruker Optics). The internal reflection element was a diamond crystal (2×2 mm) with an aperture angle of 45°, yielding a single internal reflection. The spectrometer was purged continuously with dried air. Spectra were obtained from 4000 cm^−1^ to 800 cm^−1^ at a resolution of 2 cm^−1^. In order to achieve a good signal-to-noise ratio, 128 scans were acquired. All measurements were conducted at 24°C. Samples were prepared by spreading 2 µl of peptide solution on a diamond crystal surface and removing the excess fluid under nitrogen flow. The film was washed three times with milliQ water, which was then removed under nitrogen flow.

### Spectral analysis

Data were processed using “Kinetics”, a program developed in our laboratory and running under MatLab. Briefly, spectra were subjected to water-vapor subtraction using a reference water vapor spectrum, and to a smoothing procedure to 4 cm^−1^. Spectra were deconvoluted using the Lorentzian deconvolution function and the Gaussian apodization function. A linear baseline was subtracted from all spectra at 1708, 1602, and 1482 cm^−1^.

### Atomic force microscopy (AFM)

AFM contact mode images were obtained at room temperature with a Nanoscope IV Multimode AFM (Veeco). Fresh mica surfaces (36 mm^2^) were glued onto steel sample discs (Veeco) with Epotek 377 (Gentec Benelux). Atomically smooth surfaces were generated by cleaving layers with adhesive tape. Peptide solution (100 µl; 0.1 mg/ml) was adsorbed onto bare mica surfaces for 1 h. The mounted samples were immediately transferred into the AFM liquid cell, while avoiding dewetting. They were then imaged with oxide-sharpened microfabricated Si_3_N_4_ cantilevers (Microlevers, Veeco) with minimal applied force (<500 pN). The spring constants of the cantilevers measured by the thermal noise method (Picoforce, Veeco) were 0.011 N/m. Images (5 µm × 5 µm) were obtained from several areas on each sample.

## Results

### Preferential formation of oligomeric, not fibrillar, species by Aβ(1–40) in the presence of Ca^2+^

**Figure 1:**
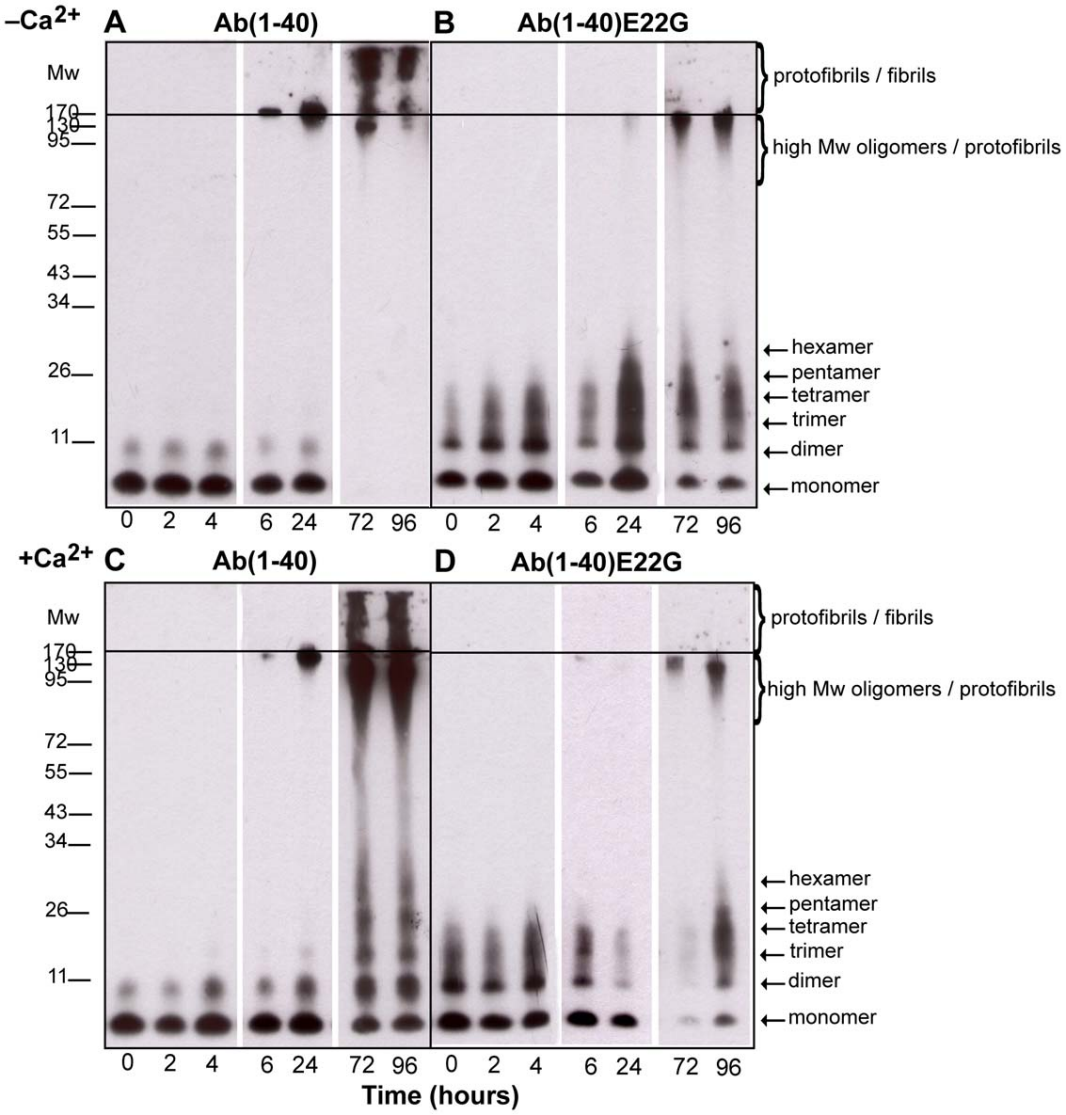
Aggregation profiles of Aβ(1–40) and Aβ(1–40)E22G by Western blot analysis. Aggregation profiles of Aβ(1–40) and Aβ(1–40)E22G during 96 h of incubation in the presence or absence of added Ca^2+^ were followed using Western blot analysis. Samples were separated using gel electrophoresis on a 12% bis-Tris gel. For each condition, samples were taken at *t* = 0, 2, 4, 6, 24, 72, and 96 h. Following the loading of 1 µg of protein sample into each lane, the membrane was probed with a mixture of monoclonal antibodies 6E10 and 4G8 that recognize residues 1–17 and 17–24, respectively. Panels A and B are representative Western blots of Aβ(1–40) and Aβ(1–40)E22G in phosphate buffer (“–Ca^2+^ condition”), respectively. Panels C and D are representative Western blots of Aβ(1–40) and Aβ(1–40)E22G in 2 mM Ca^2+^ (“+Ca^2+^ condition”), respectively. At least four separate experiments were carried out to confirm these results. All images were taken from a single 96-h experimental procedure.

Samples of Aβ(1–40) and Aβ(1–40)E22G were prepared and incubated for 96 h in either the presence or the absence of 2 mM Ca^2+^. At time points corresponding to *t* = 0, 2, 4, 6, 24, 72, and 96 h, samples were analyzed by electrophoresis in SDS-free polyacrylamide gel (0.1% SDS was present in the migration buffer) and imaged by Western blotting (Fig. 1). Over time, the aggregation pattern of Aβ(1–40) showed striking differences in the presence and in the absence of added Ca^2+^ (Fig. 1A, C). Whereas after 24 h the range of oligomeric species in the samples with and without added Ca^2+^ was almost indistinguishable, at 72 h we observed a wide range of species in the presence of Ca^2+^, but not in its absence. At 72 h and 96 h, Aβ(1–40) in the presence of Ca^2+^ contained monomers and oligomers whose molecular weights ranged from those consistent with dimers (around 8 kDa) to hexamers (Fig. 1C, two last lanes). Additional streaks in the same lanes suggested the presence of oligomers of even higher molecular weight, though they were not clearly identified. In the same samples, protofibrils and apparently some fibrils were also detectable at the top portion of the gel. It was difficult to differentiate between these aggregates because of the low resolution in this part of the gel and their low electrophoretic mobility. Fibrils, because of their extremely high molecular weight, do not penetrate into the separating part of the polyacrylamide gel; thus, when present, they appear as smears in the stacking portion of the gel. In the absence of added Ca^2+^, Aβ(1–40) molecules had aggregated to such an extent that we were able to detect only bands with low electrophoretic mobility corresponding mainly to high-molecular-weight oligomers, protofibrils and fibrils, located in or near the stacking part of the polyacrylamide gel (Fig.1A, two last lanes).

In contrast to Aβ(1–40), samples of Aβ(1–40)E22G that were incubated under the same conditions as the Aβ(1–40) samples showed no differences in their aggregation profiles in the presence and absence of added Ca^2+^ (Fig. 1B, D). Moreover, already at *t* = 0 h we observed oligomers ranging from monomers to tetramers that were not present in Aβ(1–40) samples at the same time point. From *t* = 24 h, we observed an increase in the population of oligomers of low molecular weight as well as the appearance of high-molecular-weight oligomers in Aβ(1–40)E22G samples, in both conditions. By *t* = 72 h and *t* = 96 h a wide range of oligomers, including protofibrils, could be seen. These findings clearly indicated that Ca^2+^ had no influence on the ability of Aβ(1–40)E22G to aggregate as expected, with the generation mainly of oligomers and protofibrils.

A comparison of the results obtained for the two peptides thus clearly showed that in the presence of Ca^2+^ Aβ(1–40), like Aβ(1–40)E22G, aggregated to produce oligomers and protofibrils, whereas in the “–Ca^2+^” condition fibrils were readily formed. The profile of oligomeric and protofibrillar species formed by Aβ(1–40) in the presence of Ca^2+^, as detected by Western analysis, was essentially the same as that of Aβ(1–40)E22G in either condition.

### Ca^2+^ inhibits formation of thioflavin T-positive Aβ(1**–**40) species but does not affect Aβ(1–40)E22G

**Figure 2:**
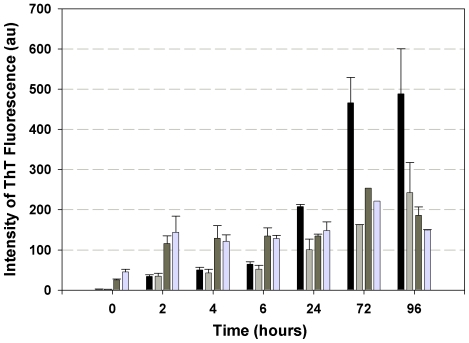
Oligomers and fibrils formation differentiated by ThT fluorescence. ThT fluorescence intensity was monitored to follow fibrillogenesis of Aβ(1–40) and Aβ(1–40)E22G in the presence and in the absence of 2 mM Ca^2+^. Black bars, Aβ(1–40) in phosphate buffer (“–Ca^2+^ condition”); light grey bars, Aβ(1–40) in 2 mM CaCl_2_; dark grey bars, Aβ(1–40)E22G in phosphate buffer; light blue bars, Aβ(1–40)E22G in CaCl_2_. Shown are averages of values obtained in four independent experiments; error bars indicating the standard error of the average.

The increase in ThT fluorescence over time was used to follow fibrillogenesis of amyloid peptides Aβ(1–40) and Aβ(1–40)E22G in solution in the presence and absence of added Ca^2+^. We found that Ca^2+^ inhibited the formation of ThT-positive species of Aβ(1–40), but had no effect on Aβ(1–40)E22G (Fig. 2).

We performed a time-course study during 96 h of incubation with or without Ca^2+^ for both peptides. In the case of Aβ(1–40), ThT fluorescence intensity did not change in either the presence or the absence of added Ca^2+^ and remained low for the first 6 h, demonstrating that after 6 h of incubation the predominant species are oligomers. This result is in agreement with the reported finding that ThT fluorescence clearly discriminates between oligomers and fibrils of Aβ [46]. After 24 h of incubation, ThT fluorescence intensity was found to be increased significantly (about three fold) in the absence of added Ca^2+^ but only slightly in its presence. After 72 and 96 h of incubation the ThT fluorescence intensity in the absence of added Ca^2+^ increased dramatically, reaching values close to 500 arbitrary units. Control samples with known fibrillar content yielded similar fluorescence values (data not shown), leading us to conclude that fibrils were the main species in our sample. This conclusion is supported by a number of studies in which the characteristic fluorescence exhibited by ThT was attributed to the binding of ThT molecules within a cavity that was present in some proteins and amyloid fibrils, but not in others [47], [48]. After 96 h of incubation of Aβ(1–40) in the presence of Ca^2+^, fluorescence intensity values remained low and were attributed to the existence of only a small population of ThT-positive species.

By contrast Aβ(1–40)E22G, both with and without Ca^2+^, exhibited stable and relatively low ThT fluorescence intensity over the course of 96 h (Fig. 2), implying either that the aggregation process is significantly slower than for Aβ(1–40) or that Aβ(1–40)E22G has a relatively high propensity to form oligomers rather than fibrils. A high tendency of Aβ(1–40)E22G to form oligomers and protofibrils has already been demonstrated in several studies [49], [19], [20]. Moreover, it was suggested that this characteristic behavior may be responsible for the marked toxicity of Aβ(1–40)E22G [21]. Individuals carrying the Arctic mutation are known to be prone to development of AD early in life [19], possibly because of the formation of oligomers and protofibrils, considered to be more toxic aggregates of Aβ peptide than fibrils.

### Oligomers formed by Aβ(1–40) in the presence of Ca ^2+^ and oligomers formed by Aβ(1–40)E22G demonstrate similar secondary structures

Secondary structures of Aβ aggregates are known to possess high β-sheet content. Using ATR−FTIR spectroscopy, our group recently showed that a characteristic signature of soluble oligomers of Aβ is an anti-parallel β-sheet conformation, whereas parallel β-sheet conformation is indicative of the presence of Aβ fibrils [50], [51]. Working with Aβ(1–42) and Aβ(1– 40), they demonstrated that in anti-parallel β-sheet structures the amide I region displays two typical components: the major component has an average spectral wavenumber at ∼1630 cm^−1^ while the minor component, about five fold weaker than the major one, is characterized by an average wavenumber at ∼1695 cm^−1^. For parallel β-sheet structures the amide I region displays only the major component of ∼1630 cm^−1^. The intensity ratio of 1695/1630 was suggested to be proportional to the percentage of anti-parallel β-strands arranged in a β-sheet [52].

**Figure 3:**
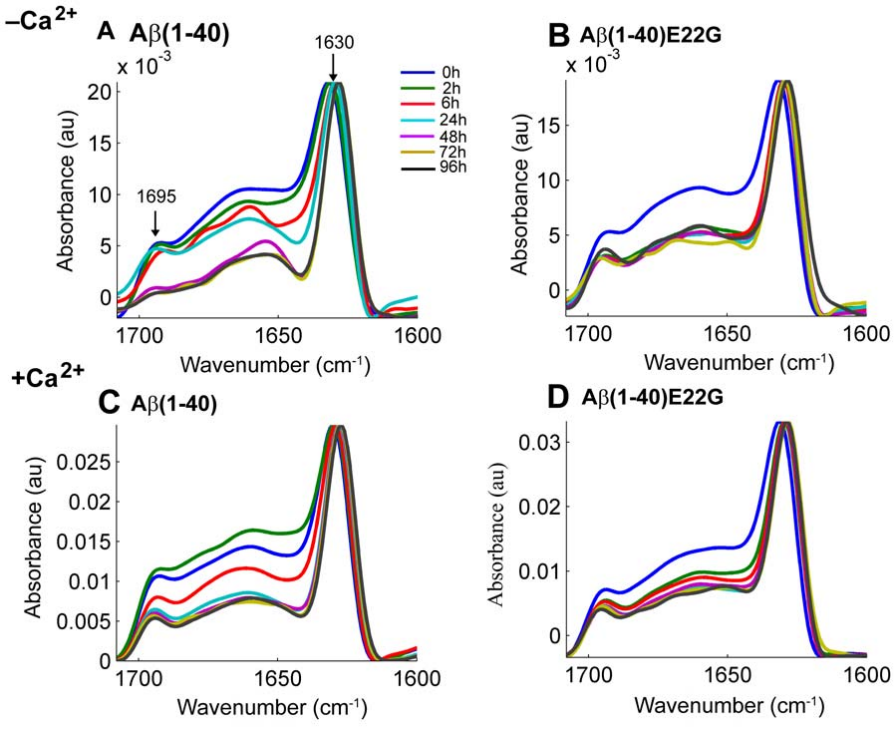
ATR-FTIR spectra of Aβ(1–40) and Aβ(1–40)E22G. FTIR spectra of Aβ(1–40) and Aβ(1–40)E22G were taken in the presence and in the absence of added Ca^2+^, showing the amide I region of the spectra (1600–1700 cm^−1^). Aliquots of 2 µl were taken from each sample at *t* = 0, 2, 6, 24, 48, 72, and 96 h (shown in blue, green, red, cyan, purple, mustard, and dark blue, respectively). The data shown here were collected in one continuous experiment and are representative of three independent trials.

Using ATR−FTIR, we studied the aggregation patterns of Aβ(1–40) and Aβ(1–40)E22G and followed the evolution of 1630-cm^−1^ and 1695-cm^−1^ peaks to assess the presence of oligomers or fibrils as a function of incubation time, based on discrimination of a β-sheet conformation. Figure 3 summarizes the results observed for each of these amyloid peptides in the presence and in the absence of added Ca^2+^. For all conditions evaluated, during the first 48 h we observed two characteristic features: one peak at ∼1695 cm^−1^ and another at ∼1630 cm^−1^ (Fig. 3, panels A–D). The presence of a peak at ∼1695 cm^−1^ in the infrared spectrum, in addition to a peak at ∼1630 cm^−1^, is characteristic of an anti-parallel β-sheet conformation, indicative of species structurally different from fibrils [51], [50]. However, for Aβ(1–40) in the absence of added Ca^2+^ we observed a significant decrease in the ∼1695 cm^−1^ peak at t = 48 h (Fig. 3A) and a shift towards lower wavenumbers and narrowing of the ∼1630 cm^−1^ peak. This shift (from 1633 cm^−1^ to 1629 cm^−1^) and narrowing were also detectable to some extent at earlier time points. This specific feature indicated formation of stable and/or long β-strands and strong hydrogen bonds, as would be expected for a stable fibrillar structure [53]. Given that the ratio of 1695/1630 is proportional to the percentage of anti-parallel arrangement of β-strands [52], we used this ratio to estimate the degree of structural change in our samples. For Aβ(1–40) in the “–Ca^2+^ condition” the 1695/1630 ratio decreased dramatically from 0.32 at *t* = 0 h to 0.08 at *t* = 96 h, meaning that there was four times less anti-parallel β-sheet structure after 96 h of incubation in the absence of added Ca^2+^. This pattern of decrease in the amount of anti-parallel β-strands in a β-sheet points to the formation of fibrillar assemblies from oligomers initially present in the sample. This result complements the outcome of the PAGE analysis and the ThT fluorescence experiments, where after the first 24 h we detected mainly oligomers whose binding affinity for ThT was low, whereas at *t* = 96 h the species observed by PAGE were mainly of high molecular weight (including fibrils), which were highly ThT positive. This result for Aβ(1–40) in phosphate buffer portrayed a dynamic process of fibrillization of the Aβ(1–40) peptide in vitro, starting with monomers and dimers, which over time were converted into fibrils. It was interesting to note that at *t* = 72 h and *t* = 96 h, no monomers, dimers, or other low-molecular-weight oligomers were visible on Western blots, indicating abundant conversion to fibrils.

The behavior of Aβ(1–40) samples in the presence of Ca^2+^ differed from their behavior in the absence of added Ca^2+^. Throughout the duration of the experiment with Aβ(1–40) in the presence of Ca^2+^ we always observed the two characteristic peaks at ∼1695 cm^−1^ and ∼1630 cm^−1^ (Fig. 2, panel C). Despite the slight decrease observed over time in the intensity of the ∼1695 cm^−1^ peak, no significant narrowing of the peak width at ∼1630 cm^−1^ was detected.

An interesting observation was that this spectral behavior of Aβ(1–40) in the presence of Ca^2+^ had the same features as that of Aβ(1–40)E22G in either the presence or the absence of Ca^2+^. Under both conditions, the peaks exhibited by Aβ(1–40)E22G at ∼1695 cm^−1^ and at ∼1630 cm^−1^ throughout the 96-h time period were characteristic of an anti-parallel β-sheet conformation (Fig. 3, panels C, B, D), and the ratio between the peaks changed only slightly, mainly after the first 2 h. In addition, no narrowing of the peak at ∼1630 cm^−1^ was detected. This finding, obtained by ATR−FTIR, showed good correlation with the results we obtained by PAGE analysis and in our ThT fluorescence experiments, all of which pointed—as expected, and in line with the published data [20], [21]—to the formation of a wide range of oligomers and possibly also protofibrils by Aβ(1–40)E22G. The similarity in aggregation patterns of Aβ(1–40) in the “+Ca^2+^ condition” and of Aβ(1–40)E22G under both conditions, as observed here by three independent techniques, raises a fundamental question concerning a possible change in the mechanism of Aβ(1–40) aggregation when Ca^2+^ is present. It seems that calcium ions promote the preferential formation of oligomers and protofibrils, diverting the otherwise favored fibrillogenesis pathway.

### Formation of Aβ(1–40) species morphologically similar to the species formed by Aβ(1–40)E22G in the presence of Ca^2+^

**Figure 4:**
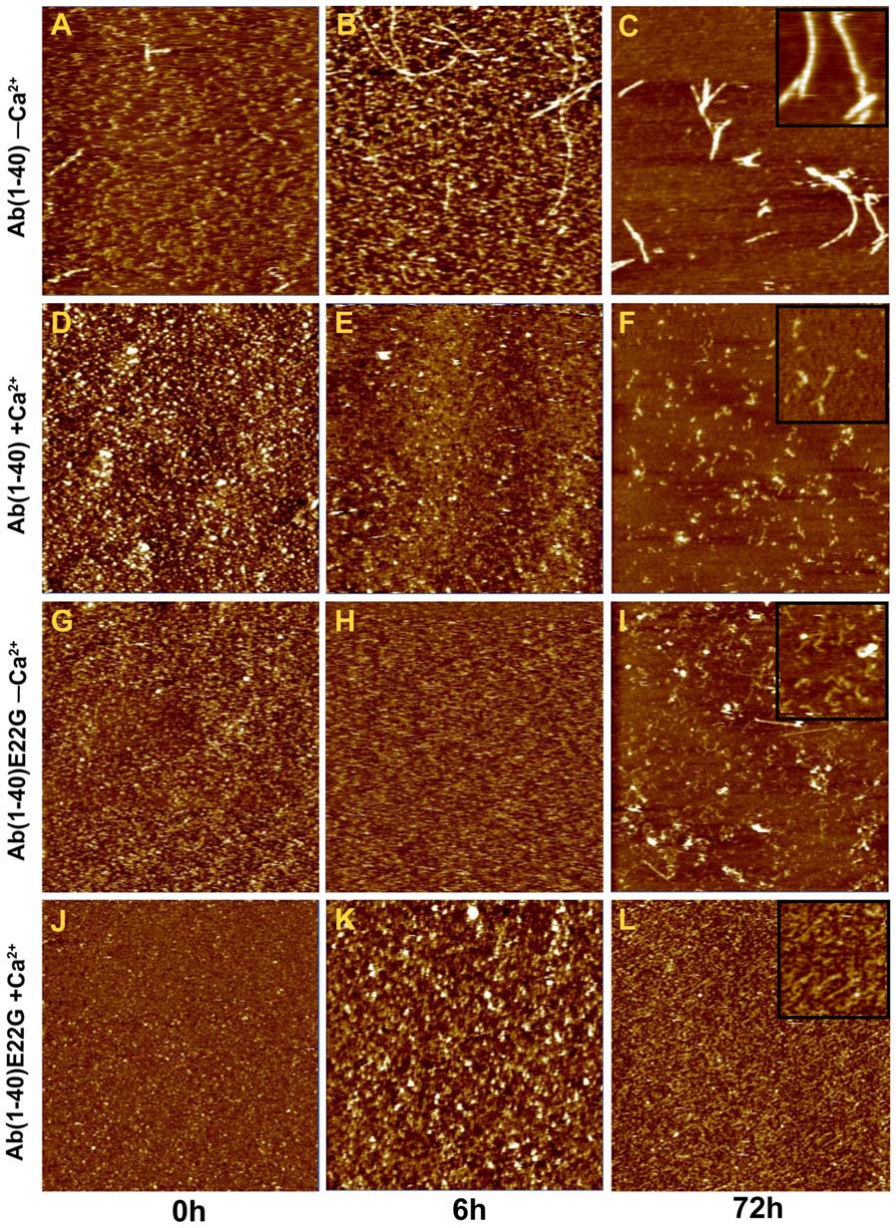
Morphological comparison of Aβ(1–40) and Aβ(1–40)E22G. Contact mode AFM images (5 µm × 5 µm, Z scale 15 nm) of Aβ(1–40) and Aβ(1–40)E22G peptides on mica, recorded either in phosphate buffer or in MOPS buffer with Ca^2+^. Samples of Aβ(1–40) and Aβ(1–40)E22G in the presence and absence of added Ca^2+^ (marked as “+Ca^2+^” or “−Ca^2+^”, respectively) at *t* = 0, 6, or 72 h. Closer views (1 µm × 1 µm, Z scale 15 nm) of oligomers, protofibrils and fibrils are shown as insets in the panel of *t* = 72 h (C, F, I, L). Images A, D, G, J were taken at *t* = 0; images B, E, H, K were taken at *t* = 6 h. Peptide concentration was the same in all samples.

Next we examined whether the oligomers formed by Aβ(1–40) in the presence of Ca^2+^ and the oligomers formed by Aβ(1–40)E22G in the presence and in the absence of added Ca^2+^ share morphological similarities. Using contact mode AFM, we followed oligomerization and fibrillogenesis of Aβ(1–40) and of Aβ(1–40)E22G, in all cases in the presence and absence of added Ca^2+^, at three time points: *t* = 0, 6, and 72 h (Fig. 4). These times were chosen because most of the differences detected by Western blot analysis, ThT fluorescence, and ATR−FTIR spectroscopy were observed after 0, 6, and 72 h of incubation.

At *t* = 0 h, samples of Aβ(1–40) and of Aβ(1–40)E22G, in all cases both in the presence and in the absence of added Ca^2+^, contained homogeneously distributed globular particles (Fig. 4, panels A, D, G, J), in agreement with previously reported data [54]. The height of the spherical aggregates was 2.8±0.3 nm (*n* = 51). Although some linear aggregates were detected in Aβ(1–40) samples in the absence of added Ca^2+^ (Fig. 4A), most of the population consisted of globular particles. Since no contribution was seen from the linear aggregates when these samples were tested with other techniques, it seems reasonable to assume that only an insignificant percentage of the peptide was present in the linear aggregate form (perhaps structurally different from fibrils) at the initial stages of incubation. Notably, this population was observed only in the Aβ(1–40) samples lacking Ca^2+^, even though all the samples received identical treatment.

A second batch of samples was imaged at *t* = 6 h. As expected, no differences in aggregate morphology were noticed between the Aβ(1–40) samples in the presence of Ca^2+^ and the Aβ(1–40)E22G samples in the presence or absence of added Ca^2+^ (Fig. 4E, H, K). All of the particles appeared to be globular, though somewhat larger than at *t* = 0 h (height 3.3±0.5 nm, *n* = 20). Evolution of the species was observed only with Aβ(1–40) in the absence of added Ca^2+^. In this case there was clear evidence of fibrillization, resulting in a large amount of string-like aggregates and a long, fibril-like species (Fig.4B). Although comparable in length (950±460 nm, *n* = 20) to previously reported fibril lengths [54], they were too thin to be categorized as fibrils. The string-like structures had become more abundant, and the fraction of spherical particles that remained was small (Fig. 4B).

The most striking differences were those observed between samples imaged at *t* = 72 h. The Aβ(1–40) samples without Ca^2+^ exhibited well-organized fibrils (Fig. 4C) whose dimensions (height 8.4±0.5 nm, *n* = 13; width 124±15 nm, *n* = 12; length 1±0.6 µm, *n* = 67) mostly correlated with those previously published for Aβ(1–40) [53]. When Aβ(1–40) was incubated in the presence of Ca^2+^ the resulting aggregates were not fibrillar, but rather spherical (height, 2.6±0.4 nm; *n* = 24) and (mostly) curvilinear (Fig. 4F) (height 3.0±0.5 nm; *n* = 28; length 250±73 nm; *n* = 40), which previous authors have referred to as oligomers and protofibrils, respectively [49]. Like the results described above and therefore in line with our expectations, Aβ(1–40) in the presence of Ca^2+^ and Aβ(1–40)E22G both in the presence and in the absence of added Ca^2+^ all formed the same oligomeric species: initially spherical particles, evolving to curvilinear structures after 72 h. These values are consistent with the data from Mastrangelo et al. [55], where the authors reported z-heights of 2–3 nm on average, for early (<1 h) oligomers and ∼2 nm for monomers of Aβ(1–42) obtained by high resolution AFM under hydrated conditions. Detailed analysis of their results revealed that for low molecular weight oligomers as well as protofibrils the z-height ranged between 2 to 4 nm. Only for some of the high molecular weight oligomers Mastrangelo et al. reported values of z-height 4–6 nm. It is likely that Aβ(1–40) and Aβ(1–42) do not form the same type of high molecular weight oligomers and the growth of Aβ(1–40) oligomers might be restricted to the lateral dimension, without causing height changes from monomers to oligomers.

In the presence of Ca^2+^, the aggregation pathway of Aβ(1–40) was clearly shifted towards formation of oligomers, and not of fibrils as occurred in the absence of added Ca^2+^. Evidently, therefore, the presence of calcium ions has a significant impact on the aggregation process of Aβ(1–40).

## Discussion

The pathogenesis of Alzheimer's disease is complex, and involves marked molecular, cellular, and physiological changes. The Ca^2+^ hypothesis, which introduced the concept of regulation by Ca^2+^ of neuronal death both in age-related and in pathogenic processes, attempts to explain how disruptions in Ca^2+^ homeostasis that continue over a prolonged period are a proximate cause of neurodegeneration in Alzheimer's disease. Numerous studies have linked Aβ to Ca^2+^ through demonstrating Ca^2+^ up-regulation by amyloid aggregates and relating Ca^2+^ dysregulation to AD-causing mutations. Accumulation of Aβ aggregates has been shown to initiate a complex pathological cascade, leading ultimately to memory alterations, cognitive impairments, and neuronal death [56]. Questions remain, however, concerning the role of early preclinical processes that predate the pathology and may enable or accelerate the aggregation of Aβ, thereby contributing to development of the disease. In particular, the possible contributory effect of normal physiological changes that take place in old age is still unknown.

In this work we tried to determine whether calcium can facilitate the formation of oligomers that might in turn be held responsible for neuronal toxicity. Using four different techniques, we showed that Aβ(1–40) forms oligomers and protofibrils in the presence of 2 mM Ca^2+^ similar to those produced by Aβ(1–40)E22G both in the presence and in the absence of added Ca^2+^ (21 µM of calcium ion were present in “−Ca^2+^ condition” buffer due to their traces in MilliQ water and in HPLC-grade buffer). We found that in the “−Ca^2+^ condition” Aβ(1–40) readily formed fibrils, which were detectable by PAGE and by ThT fluorescence analysis as well as by FTIR, and differences in secondary structures were observed between oligomers and fibrils. Moreover, AFM imaging clearly revealed morphological similarities between oligomers of Aβ(1–40) and of Aβ(1–40)E22G, all of which were spherical or curvilinear in shape. Aβ(1–40)E22G has been shown to form oligomers and protofibrils in vivo and to cause early and severe signs of AD in mice [57]. It remains an open question whether the morphological similarity of oligomers of Aβ(1–40) formed in the presence of Ca^2+^ to oligomers of Aβ(1–40)E22G formed also in the absence of added Ca^2+^ implies that they have similar toxic effects.

**Figure 5:**
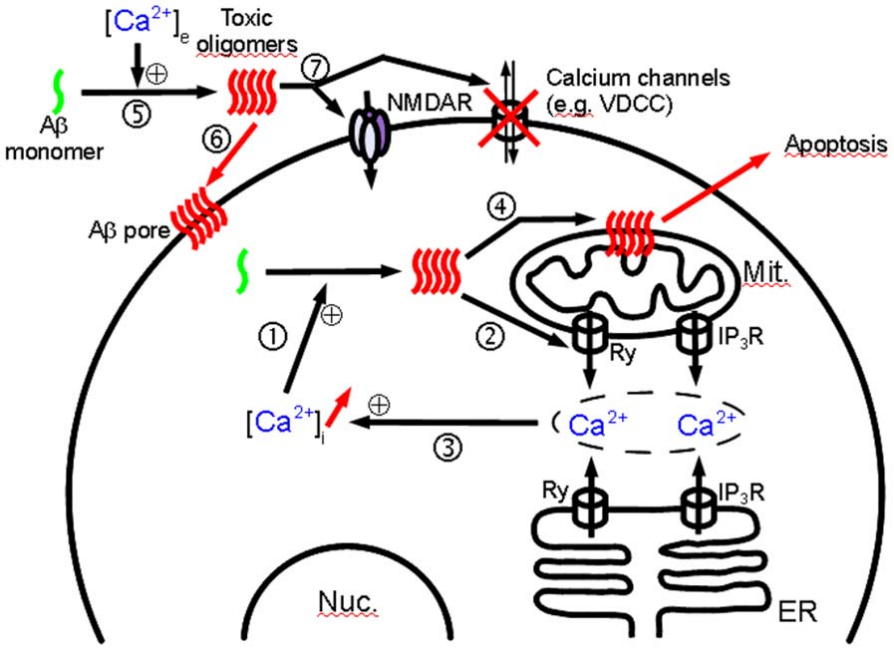
Potential interplay between Aβ oligomers, Ca^2+^, and a target cell in the initial stages of Alzheimer's disease. (1) Age-related increase in [Ca^2+^]_i_ promotes oligomerization of intracellular Aβ. (2) Disruption of Ca^2+^ homeostasis by oligomers, by either binding to or modulating the activity of a number of receptors such as ryanodine (Ry) and inositol triphosphate (IP_3_R) [25]. (3) Increase in [Ca^2+^]_i_. These three steps might form an inimical cycle leading to increases in both cytosolic calcium and Aβ oligomer concentrations. (4) Aβ oligomers disrupt intracellular membranes, leading to apoptosis [34], [41], [64]. (5) Extracellular calcium concentration ([Ca^2+^]_e_) promotes oligomerization of extracellular Aβ. (6) Oligomers form nonspecific pores in the plasma membrane, disturbing cellular integrity and leading to apoptosis [65]. (7) Aβ oligomers can interact and impair calcium channels at the membrane surface, opening calcium importers and blocking calcium exporters such as the voltage-dependent calcium channel [66]. Aβ oligomers can affect surface expression of N-methyl-D-aspartate receptors (NMDARs) [67], may increase [68] or decrease the conductance [69], and facilitate long-term synaptic depression by disrupting neuronal glutamate uptake [70].

Previous studies of the role of Ca^2+^ in Aβ aggregation have revealed acceleration of Aβ(1–42) fibril formation in the presence of Ca^2+^ [58], [59], but without acceleration in the kinetics of Aβ(1–40) fibrillization [59]. In the present study, however, we focused on the role of Ca^2+^ in the formation of oligomers, rather than in fibril formation. Our experiments demonstrated that 2 mM Ca^2+^ catalyze the formation of oligomers of Aβ(1–40), whereas in the absence of added Ca^2+^ mostly fibrils were formed. To the best of our knowledge, this is the first time that Ca^2+^ has been shown to induce Aβ(1–40) to form oligomers. We conducted our present experiments with 2 mM added CaCl_2_ and 100 µM Aβ. Ca^2+^ concentrations in the neuronal cytosol vary from hundreds of nanomolars to micromolars, which is consistent with the concentration of calcium traces in the “−Ca^2+^ buffer”. Higher cytosolic calcium concentration may occur for prolonged periods during slow after-polarization current of ER after Ca^2+^ release from intracellular stores during LTP induction [60]. The cytosolic Ca^2+^ load is an important factor in regulating the size of mitochondrial Ca^2^+ stores [60], and during neuronal activity, which includes increases in cytosolic Ca^2+^, mitochondria can take up significant loads of Ca^2+^ [61]. In addition, 2 mM Ca^2+^ is close to the concentration in the extracellular space, where formation of oligomers may be initiated and from where they might subsequently exert their toxic effect on the plasma membrane [62], [63]. Comparison of experiments carried out at 20 µm and 2 mM calcium suggests that extracellular calcium promotes oligomerization of Aβ(1–40). From additional information obtained by measuring residual calcium in the “−Ca^2+^” buffer it is tempting to conclude that the intracellular concentration of calcium does not promote oligomerization. It remains however, that the intracellular Aβ peptides and calcium concentrations are difficult to evaluate and the existence of an intracellular effect can not be rejected at this stage. Based on the previously published data (as outlined in the Introduction) as well as our present findings, we schematically summarize the potential effects of both extra- and intracellular calcium ions on Aβ and cells (Fig. 5).

To conclude, our results show that the formation of Aβ(1–40) oligomers is induced in the presence of 2 mM Ca^2+^, whereas in the presence of as little as 20 µM Ca^2+^ Aβ(1–40) undergoes fibrillogenesis. The mechanism of Ca^2+^-induced Aβ(1–40) aggregation is currently under investigation. Nevertheless, the above finding might constitute the missing link that connects early dysregulation in Ca^2+^ signaling to later onset of pathological and/or cognitive symptoms characteristic of AD. In their recent review focusing on intracellular Aβ production and its assembly states, LaFerla et al. [63] suggested that the buildup of intracellular Aβ might be an early event in the pathogenesis of AD as well as of Down syndrome. Taking that notion further, we contemplate that the early event in AD pathology might be the aggregation of intracellular Aβ in response to an increase in [Ca^2+^]_i_ as a result of natural aging processes. These early aggregates could in turn exert their toxic effect to alter Ca^2+^ signaling, which may account for the progressive decline in memory and the increase in neuronal cell apoptosis that occurs during AD. This may constitute a possible reason why in old age, when calcium imbalance is pronounced, the probability of developing AD is increased. This may also offer an alternative approach to prevention and treatment strategies for this disease, targeting mechanistic causes rather than late-stage symptoms.
